# Supplementary material for: Differential Detection of Alternaria alternata Haplotypes Isolated from Carya illinoinensis Using PCR-RFLP Analysis of Alt a1 Gene Region
Source: Genes (Basel). 2023 May 20;14(5):1115. doi: 10.3390/genes14051115 (PMC10218372; doi:10.3390/genes14051115)
Supplement: Supplementary file 1 [file genes-14-01115-s001.zip › Supplementary Figure.pdf]

Supplementary Figure S1

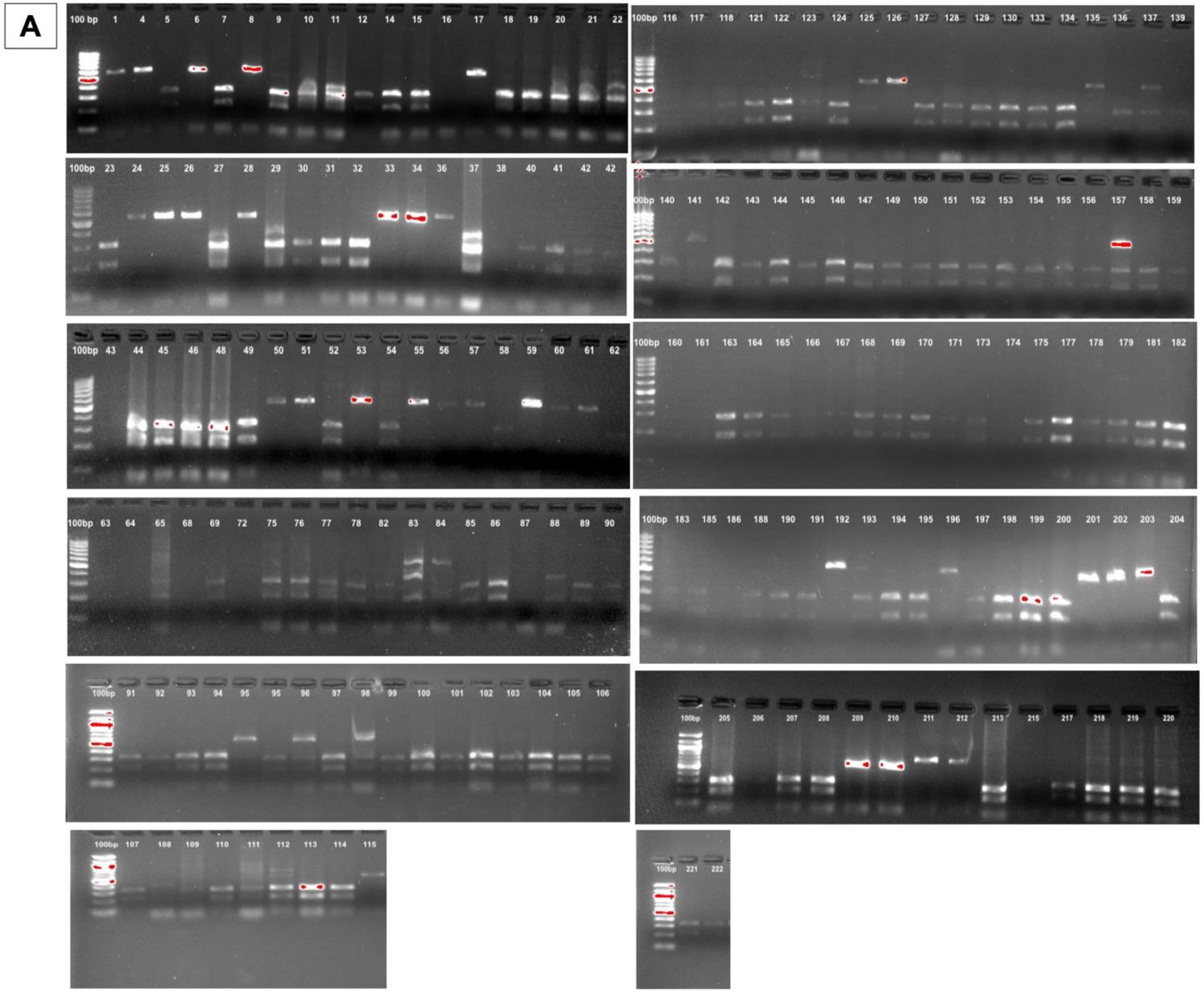

Supplementary Figure S1A. RFLP profiles of *Alt a1* DNA PCR products of 222 *Alternata alternata* isolates using *Hae*III endonuclease digestion, Lane 1: (100 bp) Molecular weight marker (Thermo Fisher Scientific, Massachusetts, USA).

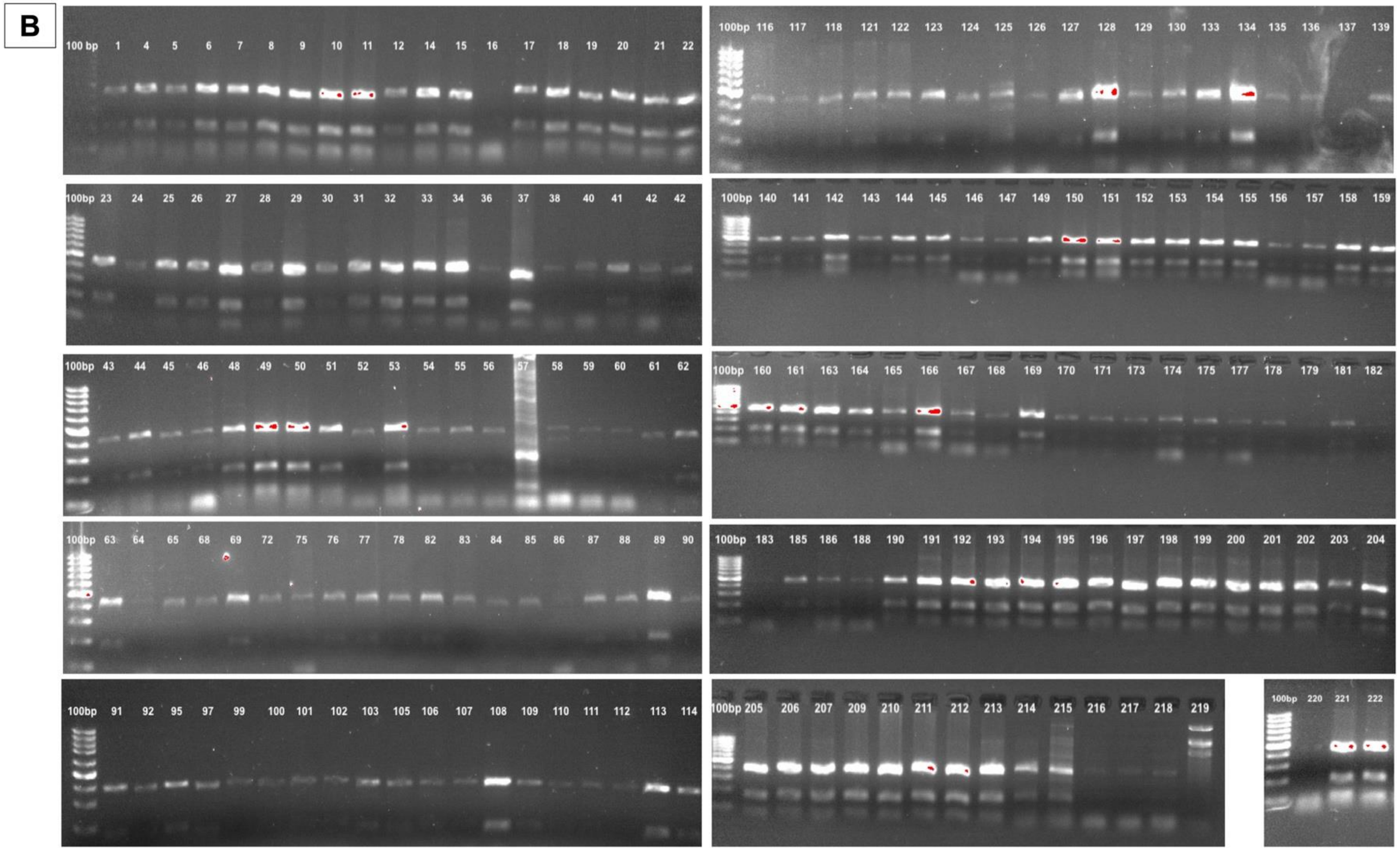

Supplementary Figure S1B. RFLP profiles of *Alt a1* DNA PCR products of 222 *Alternata alternata* isolates using *Hinf*I endonuclease digestion, Lane 1: (100 bp) Molecular weight marker (Thermo Fisher Scientific, Massachusetts, USA).
