# Supplementary material for: Differential Detection of Alternaria alternata Haplotypes Isolated from Carya illinoinensis Using PCR-RFLP Analysis of Alt a1 Gene Region
Source: Genes (Basel). 2023 May 20;14(5):1115. doi: 10.3390/genes14051115 (PMC10218372; doi:10.3390/genes14051115)
Supplement: Supplementary file 1 [file genes-14-01115-s001.zip › Supplementary Table.pdf]

Supplementary Table S1. Details on information of *Alternaria alternata* isolates used in this study (2017 -2019)

Number	*A. alternata* isolates	Location	Coordinates	Pecan Substrates
1	CGJM3112	Gauteng	25°35'13.6"S 28°33'31.6"E	Symptomatic Nuts-in-shuck
2	CGJM3028	Gauteng	25°35'13.6"S 28°33'31.6"E	Symptomatic Leaves
3	CGJM3026	Gauteng	25°35'13.6"S 28°33'31.6"E	Symptomatic Leaves
4	CGJM3125	Gauteng	25°35'13.6"S 28°33'31.6"E	Symptomatic Leaves
5	CGJM3136	Gauteng	25°35'13.6"S 28°33'31.6"E	Symptomatic Shoots
6	CGJM3097	Gauteng	25°35'13.6"S 28°33'31.6"E	Symptomatic Leaves
7	CGJM3105	Limpopo	24°40'40.5"S 28°29'55.6"E	Symptomatic Leaves
8	CGJM3053	Limpopo	24°40'40.5"S 28°29'55.6"E	Symptomatic Nuts-in-shuck
9	CGJM3119	Limpopo	24°40'40.5"S 28°29'55.6"E	Symptomatic Shoots
10	CGJM3057	Limpopo	24°40'40.5"S 28°29'55.6"E	Symptomatic Shoots
11	CGJM2955	Limpopo	24°40'40.5"S 28°29'55.6"E	Symptomatic Shoots
12	CGJM3109	Limpopo	24°40'40.5"S 28°29'55.6"E	Symptomatic Leaves
13	CGJM3126	Limpopo	24°40'40.5"S 28°29'55.6"E	Symptomatic Leaves
14	CGJM3120	Limpopo	24°40'40.5"S 28°29'55.6"E	Symptomatic Leaves
15	CGJM3128	Limpopo	24°40'40.5"S 28°29'55.6"E	Symptomatic Leaves
16	CGJM3154	Limpopo	24°40'40.5"S 28°29'55.6"E	Symptomatic Leaves
17	CGJM3067	Limpopo	24°40'40.5"S 28°29'55.6"E	Symptomatic Leaves
18	CGJM3091	Limpopo	24°40'40.5"S 28°29'55.6"E	Symptomatic Leaves
19	CGJM3084	Limpopo	24°40'40.5"S 28°29'55.6"E	Symptomatic Leaves
20	CGJM3160	Limpopo	24°40'40.5"S 28°29'55.6"E	Symptomatic Leaves
21	CGJM3123	Limpopo	24°40'40.5"S 28°29'55.6"E	Symptomatic Leaves
22	CGJM3124	Limpopo	S 23 05'12.1" E 030 10'50.0"	Symptomatic Leaves
23	CGJM3064	Limpopo	S 23 05'12.1" E 030 10'50.0"	Symptomatic Leaves
24	CGJM3070	Limpopo	S 23 05'12.1" E 030 10'50.0"	Symptomatic Leaves
25	CGJM3061	Limpopo	S 23 05'06.3 E 030 11 05.6"	Symptomatic Leaves
26	CGJM3017	Limpopo	S 23 05'06.3 E 030 11 05.6"	Symptomatic Leaves
27	CGJM2985	Limpopo	S 23 05'06.3 E 030 11 05.6"	Symptomatic Leaves
28	CGJM3005	Limpopo	S 23 05'06.3 E 030 11 05.6"	Symptomatic Leaves
29	CGJM3047	Limpopo	24°25'42.1"S 28°35'41.0"E	Symptomatic Shoots
30	CGJM3111	Limpopo	24°25'42.1"S 28°35'41.0"E	Symptomatic Shoots
31	CGJM2965	Limpopo	24°25'42.1"S 28°35'41.0"E	Symptomatic Leaves
32	CGJM3114	Limpopo	24°25'42.1"S 28°35'41.0"E	Symptomatic Leaves
33	CGJM3101	Limpopo	24°25'42.1"S 28°35'41.0"E	Symptomatic Leaves
34	CGJM3040	Limpopo	24°25'42.1"S 28°35'41.0"E	Symptomatic Leaves
35	CGJM2978	Limpopo	24°25'42.1"S 28°35'41.0"E	Symptomatic Leaves
36	CGJM3051	Limpopo	24°25'42.1"S 28°35'41.0"E	Symptomatic Leaves
37	CGJM3024	Limpopo	24°25'42.1"S 28°35'41.0"E	Symptomatic Leaves
38	CGJM3050	Limpopo	24°25'42.1"S 28°35'41.0"E	Symptomatic Leaves
39	CGJM3100	Limpopo	24°25'42.1"S 28°35'41.0"E	Symptomatic Leaves
40	CGJM3099	Limpopo	24°25'42.1"S 28°35'41.0"E	Symptomatic Leaves
41	CGJM2959	Limpopo	24°25'42.1"S 28°35'41.0"E	Symptomatic Leaves

42	CGJM2958	Limpopo	24°25'42.1"S 28°35'41.0"E	Symptomatic Leaves
43	CGJM3027	Limpopo	24°25'42.1"S 28°35'41.0"E	Symptomatic Leaves
44	CGJM3108	Limpopo	29°36'59.8"S 30°27'53.6"E	Symptomatic Nuts-in-shuck
45	CGJM3132	Limpopo	29°36'59.8"S 30°27'53.6"E	Symptomatic Shoots
46	CGJM3138	Limpopo	29°36'59.8"S 30°27'53.6"E	Symptomatic Leaves
47	CGJM2954	Limpopo	29°36'59.8"S 30°27'53.6"E	Symptomatic Leaves
48	CGJM2989	Limpopo	29°36'59.8"S 30°27'53.6"E	Symptomatic Leaves
49	CGJM3069	Limpopo	29°36'59.8"S 30°27'53.6"E	Symptomatic Leaves
50	CGJM2964	Limpopo	29°36'59.8"S 30°27'53.6"E	Symptomatic Leaves
51	CGJM2984	Limpopo	29°36'59.8"S 30°27'53.6"E	Symptomatic Leaves
52	CGJM3062	Limpopo	29°36'59.8"S 30°27'53.6"E	Symptomatic Leaves
53	CGJM3063	Limpopo	29°36'59.8"S 30°27'53.6"E	Symptomatic Leaves
54	CGJM3033	Limpopo	29°36'59.8"S 30°27'53.6"E	Symptomatic Leaves
55	CGJM3127	Limpopo	29°36'59.8"S 30°27'53.6"E	Symptomatic Leaves
56	CGJM2976	Limpopo	29°36'59.8"S 30°27'53.6"E	Symptomatic Leaves
57	CGJM3016	Limpopo	29°36'59.8"S 30°27'53.6"E	Symptomatic Leaves
58	CGJM3092	Limpopo	29°36'59.8"S 30°27'53.6"E	Symptomatic Leaves
59	CGJM1A	Limpopo	29°36'59.8"S 30°27'53.6"E	Symptomatic Leaves
60	CGJM1B	Limpopo	29°36'59.8"S 30°27'53.6"E	Symptomatic Leaves
61	CGJM1C	Limpopo	24°40'40.5"S 28°29'55.6"E	Symptomatic Shoots
62	CGJM1D	Limpopo	24°25'42.1"S 28°35'41.0"E	Symptomatic Leaves
63	CGJM1E	Limpopo	24°25'42.1"S 28°35'41.0"E	Symptomatic Leaves
64	CGJM1F	Limpopo	29°36'59.8"S 30°27'53.6"E	Symptomatic Leaves
65	CGJM2979	Kwazulu-Natal	S 28 55'50.9" E 030 23'20.4"	Symptomatic Leaves
66	CGJM3161	Kwazulu-Natal	S 28 55'50.9" E 030 23'20.4"	Non-symptomatic Leaves
67	CGJM2986	Kwazulu-Natal	S 28 51'01.7" E 030 06'01.5"	Symptomatic Nuts-in-shuck
68	CGJM3032	Kwazulu-Natal	S 28 51'01.7" E 030 06'01.5"	Symptomatic Nuts-in-shuck
69	CGJM3042	Kwazulu-Natal	S 28 51'29.4" E 030 05'21.3"	Symptomatic Nuts-in-shuck
70	CGJM3079	Kwazulu-Natal	S 27 36'39.3" E 031 28'55.2"	Symptomatic Shoots
71	CGJM3082	Kwazulu-Natal	S 27 23'17.4" E 031 49'15.4"	Symptomatic Shoots
72	CGJM3038	Mpumalanga	S 25 25'41.3" E 030 56'15.2"	Symptomatic Leaves
73	CGJM2980	Mpumalanga	S 25 27'13.1" E 030 56'40.8"	Symptomatic Leaves
74	CGJM1I	Mpumalanga	S 25 27'13.1" E 030 56'40.8"	Symptomatic Leaves
75	CGJM3162	Eastern Cape	S 32 01'42.4" E 025 32'52.7"	Symptomatic Nuts-in-shuck
76	CGJM3085	Eastern Cape	S 32 01'42.4" E 025 32'52.7"	Symptomatic Shoots
77	CGJM3083	Eastern Cape	S 32 01'42.4" E 025 32'52.7"	Non-symptomatic Leaves
78	CGJM3093	Eastern Cape	S 32 01'42.4" E 025 32'52.7"	Non-symptomatic Leaves
79	CGJM3049	Eastern Cape	S 32 01'42.4" E 025 32'52.7"	Symptomatic Leaves
80	CGJM3037	Eastern Cape	S 32 11'57.5" E 025 38'20.9"	Symptomatic Leaves
81	CGJM3052	Eastern Cape	S 32 11'57.5" E 025 38'20.9"	Symptomatic Leaves
82	CGJM3048	Eastern Cape	S 32 11'57.5" E 025 38'20.9"	Symptomatic Leaves
83	CGJM3163	Eastern Cape	S 32 11'57.5" E 025 38'20.9"	Symptomatic Leaves
84	CGJM3115	Eastern Cape	S 32 11'57.5" E 025 38'20.9"	Symptomatic Leaves
85	CGJM3131	Eastern Cape	S 32 11'57.5" E 025 38'20.9"	Symptomatic Leaves
86	CGJM3164	Eastern Cape	S 32 11'57.5" E 025 38'20.9"	Symptomatic Leaves
87	CGJM3165	Eastern Cape	S 32 11'57.5" E 025 38'20.9"	Symptomatic Leaves
88	CGJM3045	Eastern Cape	S 32 11'57.5" E 025 38'20.9"	Symptomatic Leaves

89	CGJM3065	Eastern Cape	S 32 11'57.5" E 025 38'20.9"	Symptomatic Leaves
90	CGJM3039	Eastern Cape	S 30 39'49.6" E 026 49'36.7"	Symptomatic Nuts-in-shuck
91	CGJM3030	Eastern Cape	S 30 39'49.6" E 026 49'36.7"	Symptomatic Leaves
92	CGJM3174	Eastern Cape	S 32 11'57.5" E 025 38'20.9"	Non-symptomatic Leaves
93	CGJM3158	Eastern Cape	S 32 42'23.5" E 026 17'16.8"	Symptomatic Leaves
94	CGJM3159	Eastern Cape	S 32 44'33.9" E 025 36'57.2"	Non-symptomatic Leaves
95	CGJM3175	Eastern Cape	S 32 44'29.3" E 025 36'15.6"	Symptomatic Shoots
96	CGJM3155	Eastern Cape	S 30 39'49.6" E 026 49'36.7"	Symptomatic Nuts-in-shuck
97	CGJM1G	Eastern Cape	S 30 39'49.6" E 026 49'36.7"	Symptomatic Leaves
98	CGJM3042	North West	S 25 47'24.5" E 027 45'53.1"	Non-symptomatic Leaves
99	CGJM3166	North West	S 25 47'22.3" E 027 45'50.3"	Non-symptomatic Leaves
100	CGJM3141	North West	S 25 13'19.1" E 027 32'02.5"	Non-symptomatic Leaves
101	CGJM3054	North West	S 25 13'19.1" E 027 32'02.5"	Non-symptomatic Leaves
102	CGJM3055	North West	S 25 13'19.1" E 027 32'02.5"	Non-symptomatic Leaves
103	CGJM3073	North West	S 25 13'19.1" E 027 32'02.5"	Non-symptomatic Leaves
104	CGJM2983	North West	S 25 13'19.1" E 027 32'02.5"	Non-symptomatic Leaves
105	CGJM2956	North West	S25 31'12.9" E 027 47'55.7"	Non-symptomatic Leaves
106	CGJM3046	North West	S 25 31'22.2" E 027 47'45.0"	Non-symptomatic Leaves
107	CGJM3034	North West	S25 31'12.9" E 027 47'55.7"	Non-symptomatic Leaves
108	CGJM3139	North West	S 25 31'22.2" E 027 47'45.0"	Non-symptomatic Leaves
109	CGJM3041	North West	S25 31'12.9" E 027 47'55.7"	Non-symptomatic Leaves
110	CGJM3029	North West	S 25 31'22.2" E 027 47'45.0"	Non-symptomatic Leaves
111	CGJM3130	North West	S 25 32'41.1" E 027 48'57.6"	Non-symptomatic Leaves
112	CGJM3071	North West	S 25 32'40.7" E 027 48'56.5"	Symptomatic Leaves
113	CGJM3072	North West	S 25 32'41.1" E 027 48'57.6"	Symptomatic Leaves
114	CGJM3168	North West	S 25 32'40.7" E 027 48'56.5"	Symptomatic Leaves
115	CGJM3104	North West	S 25 32'41.1" E 027 48'57.6"	Symptomatic Leaves
116	CGJM2977	North West	S 25 32'41.1" E 027 48'57.6"	Symptomatic Leaves
117	CGJM3096	North West	S 25 32'40.7" E 027 48'56.5"	Non-symptomatic Leaves
118	CGJM3068	North West	S 27 14'27.4" E 026 09'39.3"	Non-symptomatic Leaves
119	CGJM3177	North West	S 27 14'27.4" E 026 09'39.3"	Symptomatic Leaves
120	CGJM3002	North West	S 27 14'27.4" E 026 09'39.3"	Symptomatic Leaves
121	CGJM3003	North West	S 27 14'27.4" E 026 09'39.3"	Symptomatic Leaves
122	CGJM3145	North West	S 27 14'27.4" E 026 09'39.3"	Symptomatic Leaves
123	CGJM2973	North West	S 27 14'27.4" E 026 09'39.3"	Symptomatic Leaves
124	CGJM2974	North West	S 27 14'27.4" E 026 09'39.3"	Symptomatic Leaves
125	CGJM2975	North West	S 27 14'27.4" E 026 09'39.3"	Symptomatic Leaves
126	CGJM3153	North West	S 27 14'27.4" E 026 09'39.3"	Symptomatic Leaves
127	CGJM3007	North West	S 27 14'27.4" E 026 09'39.3"	Symptomatic Leaves
128	CGJM3169	North West	S 27 14'27.4" E 026 09'39.3"	Symptomatic Leaves
129	CGJM3008	North West	S 27 14'27.4" E 026 09'39.3"	Symptomatic Leaves
130	CGJM3009	North West	S 27 14'27.4" E 026 09'39.3"	Symptomatic Leaves
131	CGJM2960	North West	S 27 14'27.4" E 026 09'39.3"	Symptomatic Leaves
132	CGJM2952	North West	S 27 14'27.4" E 026 09'39.3"	Non-symptomatic Leaves
133	CGJM2961	North West	S 27 14'27.4" E 026 09'39.3"	Non-symptomatic Leaves
134	CGJM2962	North West	S 27 14'27.4" E 026 09'39.3"	Non-symptomatic Leaves
135	CGJM2963	North West	S 27 14'27.4" E 026 09'39.3"	Non-symptomatic Leaves

136	CGJM2953	North West	S 27 14'27.4" E 026 09'39.3"	Non-symptomatic Leaves
137	CGJM2970	North West	S 27 14'27.4" E 026 09'39.3"	Non-symptomatic Leaves
138	CGJM3129	North West	S 26 24'01.0" E 026 12' 32.6"	Non-symptomatic Leaves
139	CGJM3001	North West	S 26 24'01.0" E 026 12' 32.6"	Symptomatic Leaves
140	CGJM2995	North West	S 26 24'01.0" E 026 12' 32.6"	Symptomatic Leaves
141	CGJM2992	North West	S 26 24'01.0" E 026 12' 32.6"	Symptomatic Leaves
142	CGJM2968	North West	S 26 24'01.0" E 026 12' 32.6"	Symptomatic Leaves
143	CGJM2971	North West	S 26 24'01.0" E 026 12' 32.6"	Symptomatic Leaves
144	CGJM2994	North West	S 26 24'01.0" E 026 12' 32.6"	Symptomatic Leaves
145	CGJM3011	North West	S 26 24'01.0" E 026 12' 32.6"	Symptomatic Leaves
146	CGJM3018	North West	S 26 24'01.0" E 026 12' 32.6"	Symptomatic Leaves
147	CGJM3014	North West	S 26 24'01.0" E 026 12' 32.6"	Symptomatic Leaves
148	CGJM3015	North West	S 26 24'01.0" E 026 12' 32.6"	Symptomatic Leaves
149	CGJM3013	North West	S 26 24'01.0" E 026 12' 32.6"	Symptomatic Leaves
150	CGJM3019	North West	S 26 24'01.0" E 026 12' 32.6"	Symptomatic Leaves
151	CGJM2990	North West	S 26 24'01.0" E 026 12' 32.6"	Symptomatic Leaves
152	CGJM2993	North West	S 26 35'05.1" E 026 33'18.8"	Symptomatic Leaves
153	CGJM3010	North West	S 26 24'01.0" E 026 12' 32.6"	Symptomatic Leaves
154	CGJM3118	North West	S 26 24'01.0" E 026 12' 32.6"	Symptomatic Leaves
155	CGJM2996	North West	S 26 35'05.1" E 026 33'18.8"	Non-symptomatic Leaves
156	CGJM2998	North West	S 26 35'05.1" E 026 33'18.8"	Non-symptomatic Leaves
157	CGJM2999	North West	S 26 35'05.1" E 026 33'18.8"	Non-symptomatic Leaves
158	CGJM3035	North West	S 26 35'05.1" E 026 33'18.8"	Symptomatic Leaves
159	CGJM2982	North West	S 26 35'05.1" E 026 33'18.8"	Symptomatic Leaves
160	CGJM3020	North West	S 26 35'05.1" E 026 33'18.8"	Symptomatic Leaves
161	CGJM3077	North West	S 26 35'05.1" E 026 33'18.8"	Symptomatic Leaves
162	CGJM3000	North West	S 26 35'05.1" E 026 33'18.8"	Symptomatic Leaves
163	CGJM3023	North West	S 26 35'05.1" E 026 33'18.8"	Non-symptomatic Leaves
164	CGJM3022	North West	S 26 35'05.1" E 026 33'18.8"	Non-symptomatic Leaves
165	CGJM3004	North West	S 26 35'05.1" E 026 33'18.8"	Symptomatic Shoots
166	CGJM3106	North West	S 26 47'35.1" E 026 14'37.9"	Symptomatic Leaves
167	CGJM3012	North West	S 26 47'35.1" E 026 14'37.9"	Symptomatic Leaves
168	CGJM3075	North West	S 26 47'35.1" E 026 14'37.9"	Symptomatic Leaves
169	CGJM3086	North West	S 26 35'05.1" E 026 33'18.8"	Non-symptomatic Leaves
170	CGJM3156	North West	S 25 57'23.7" E 027 24'49.8"	Non-symptomatic Leaves
171	CGJM3152	North West	S 25 57'23.7" E 027 24'49.8"	Non-symptomatic Leaves
172	CGJM3157	North West	S 25 46'47.7" E 027 45'43.3"	Non-symptomatic Leaves
173	CGJM3116	North West	S 25 46'47.7" E 027 45'43.3"	Non-symptomatic Leaves
174	CGJM3133	North West	S 25 47'41.0" E 027 45'40.7"	Non-symptomatic Leaves
175	CGJM3151	North West	S 25 47'41.0" E 027 45'40.7"	Non-symptomatic Leaves
176	CGJM3171	North West	S 25 47'06.7" E 027 44'53.6"	Symptomatic Nuts-in-shuck
177	CGJM3172	North West	S 25 13'19.1" E 027 32'02.5"	Symptomatic Leaves
178	CGJM3107	North West	S 25 13'19.1" E 027 32'02.5"	Non-symptomatic Leaves
179	CGJM3135	North West	S 25 13'19.1" E 027 32'02.5"	Non-symptomatic Leaves
180	CGJM3088	North West	S 26 35'05.1" E 026 33'18.8"	Symptomatic Leaves
181	CGJM3094	North West	S 26 35'05.1" E 026 33'18.8"	Symptomatic Leaves
182	CGJM3176	North West	S 26 51'04.6" E 026 13'40.1"	Symptomatic Leaves

183	CGJM3081	North West	S 26 35'05.1" E 026 33'18.8"	Symptomatic Leaves
184	CGJM3143	North West	S 26 35'05.1" E 026 33'18.8"	Symptomatic Leaves
185	CGJM1H	North West	S 26 35'05.1" E 026 33'18.8"	Non-symptomatic Leaves
186	CGJM3170	Free State	S 26 53'17.2" E 027 23'23.9"	Non-symptomatic Leaves
187	CGJM3117	Free State	S 26 53'17.2" E 027 23'23.9"	Non-symptomatic Leaves
188	CGJM3146	Free State	S 26 53'17.2" E 027 23'23.9"	Non-symptomatic Leaves
189	CGJM3134	Free State	S 26 51'48.9" E 027 17'45.6"	Symptomatic Nuts-in-shuck
190	CGJM3150	Free State	S 26 51'48.9" E 027 17'45.6"	Symptomatic Nuts-in-shuck
191	CGJM3149	Free State	S 26 51'48.9" E 027 17'45.6"	Symptomatic Nuts-in-shuck
192	CGJM3173	Free State	S 26 51'39.4" E 027 17'52.6"	Symptomatic Leaves
193	CGJM3148	Free State	S 26 51'39.4" E 027 17'52.6"	Symptomatic Leaves
194	CGJM3147	Free State	S 26 51'39.4" E 027 17'52.6"	Symptomatic Leaves
195	CGJM3535	Northern Cape	S 27 39'53.1" E 024 44'09.4"	Symptomatic Leaves
196	CGJM3536	Northern Cape	S 27 40'41.4" E 024 46'42.6"	Non-symptomatic Leaves
197	CGJM3537	Northern Cape	S 27 39'53.1" E 024 44'09.4"	Symptomatic Nuts-in-shuck
198	CGJM3538	Northern Cape	S 27 40'41.4" E 024 46'42.6"	Symptomatic Nuts-in-shuck
199	CGJM3539	Northern Cape	S 29 10'49.0" E 023 43'28.3"	Non-symptomatic Leaves
200	CGJM3540	Northern Cape	S 29 10'49.0" E 023 43'28.3"	Non-symptomatic Leaves
201	CGJM3541	Northern Cape	S 29 08'53.7" E 023 42'29.7"	Symptomatic Leaves
202	CGJM3542	Northern Cape	S 29 08'53.7" E 023 42'29.7"	Symptomatic Leaves
203	CGJM3543	Northern Cape	S 29 00'30.0" E 023 52'03.9"	Non-symptomatic Leaves
204	CGJM3544	Northern Cape	S 29 01'04.2" E 023 53'03.3"	Symptomatic Leaves
205	CGJM3545	Northern Cape	S 29 00'55.2" E 023 52'44.6"	Symptomatic Nuts-in-shuck
206	CGJM3546	Northern Cape	S 29 00'51.5" E 023 52'41.2"	Symptomatic Shoots
207	CGJM3547	Northern Cape	S 29 35'24.0" E 022 54'07.9"	Non-symptomatic Leaves
208	CGJM3548	Northern Cape	S 29 35'24.0" E 022 54'07.9"	Symptomatic Leaves
209	CGJM3549	Northern Cape	S 29 35'24.0" E 022 54'07.9"	Symptomatic Nuts-in-shuck
210	CGJM3550	Northern Cape	S 29 39'32.3" E 022 46'31.8"	Non-symptomatic Leaves
211	CGJM3551	Northern Cape	S 29 39'32.3" E 022 46'31.8"	Symptomatic Leaves
212	CGJM3552	Northern Cape	S 29 39'32.3" E 022 46'31.8"	Symptomatic Shoots
213	CGJM3553	Northern Cape	S 29 34'20.5" E 022 51'31.2"	Symptomatic Leaves
214	CGJM3554	Northern Cape	S 29 48'49.5 E 024 24'34.3"	Symptomatic Leaves
215	CGJM3555	Northern Cape	S 29 48'49.5 E 024 24'34.3"	Symptomatic Leaves
216	CGJM3556	Northern Cape	S 29 50'26.3" E 024 22'52.3"	Non-symptomatic Leaves
217	CGJM3557	Northern Cape	S 29 50'26.3" E 024 22'52.3"	Non-symptomatic Leaves
218	CGJM3558	Northern Cape	S 29 50'26.3" E 024 22'52.3"	Symptomatic Leaves
219	CGJM3559	Northern Cape	S 29 50'26.3" E 024 22'52.3"	Symptomatic Nuts-in-shuck
220	CGJM3560	Northern Cape	S 27 54'20.7" E 024 51'31.8"	Symptomatic Shoots
221	CGJM3561	Northern Cape	S 27 54'20.7" E 024 51'31.8"	Symptomatic Shoots
222	CGJM3562	Northern Cape	S 28 30'58.1" E 021 43'38.5"	Symptomatic Leaves

Supplementary Table S2. *Alternaria* isolates used for phylogenetic analysis in this study and their GenBank accession number (Woudenberg et al 2015)

Species name and strain number[1,2]	Locality, host / substrate	GenBank accession numbers
		Alt a 1
Alternaria alstroemeriae		
CBS 118808; E.G.S. 50.116R	USA, Alstroemeria sp.	KP123845
Alternaria alternata		
CBS 106.24; E.G.S. 38.029; ATCC 13963 (A. maliT)	USA, Malus sylvestris	KP123847
CBS 104.26	Unknown, unknown	KP123848
CBS 107.27; ATCC 24463; QM 1736 (A. citri)	USA, Citrus limonium	KP123849
CBS 154.31; IHEM 3320	USA, Staphylea trifolia	KP123851
CBS 103.33; E.G.S. 35.182; IHEM 3319 (A. soliaegyptiacaT)	Egypt, soil	KP123852
CBS 106.34; E.G.S. 06.198; DSM 62019; MUCL 10030 (A. liniT)	Unknown, Linum usitatissimum	KP123853
CBS 102.47; E.G.S. 02.062 (A. citriR)	USA, Citrus sinensis	KP123855
CBS 174.52; E.G.S. 39.1613; IMI 068086; QM 1278	USA, Anemone occidentalis	KP123856
CBS 175.52; E.G.S. 35.1619; IMI 068085; QM 1277	USA, Juncus mertensianus	KP123857
CBS 107.53; DSM 3187; IFO 5778 (A. kikuchiana)	Japan, Pyrus pyrifolia	KP123858
CBS 686.68; LCP 1988 (A. tenuissima)	Sahara, desert sand	KP123859
CBS 612.72; DSM 62012 (A. cinerariae)	Germany, Senecio cineraria	KP123861
CBS 267.77 (A. citri)	USA, Citrus paradisi	KP123864
CBS 603.78; E.G.S. 30.134; QM 9553	USA, air	KP123865
CBS 175.80 (A. septorioides)	Italy, unknown	KP123866
CBS 192.81 (A. citri)	Egypt, Citrus sinensis	KP123867
CBS 620.83; ATCC 15052 (A. tenuissima)	USA, Nicotiana tabacum	KP123868
CBS 194.86; E.G.S. 04.090; QM 1347 (A. pulvinifungicolaT)	USA, Quercus sp.	KP123869
CBS 195.86; E.G.S. 36.172; DAOM 185214 (A. angustiovoideaT)	Canada, Euphorbia esula	JQ646398
CBS 447.86 (A. malvae)	Marocco, Malva sp.	JQ646397
CBS 479.90; E.G.S. 29.028 (A. pellucidaT)	Japan, Citrus unshiu	KP123870
CBS 595.93 (A. rhadinaT)	Japan, Pyrus pyrifolia	JQ646399
CBS 877.95 (A. tenuissima)	India, human, sinusitis	KP123871
CBS 880.95; IMI 292915 (A. tenuissima)	Belgium, Fragaria vesca	np
CBS 965.95; IMI 289679 (A. tenuissima)	India, Triticum sp.	KP123872
CBS 966.95; IMI 79630 (A. tenuissima)	India, Solanum lycopersicum	KP123873
CBS 806.96	Papua New Guinea, Cyperaceae	KP123874
CBS 916.96; E.G.S. 34.016; CBS 110977; CBS 115616; IMI 254138T	India, Arachis hypogaea	AY563301
CBS 918.96; E.G.S. 34.015; IMI 255532 (A. tenuissimaR)	UK, Dianthus chinensis	AY563302
CBS 911.97; IMI 056271 (A. tenuissima)	India, Artemisia brevifolia	KP123875
CBS 639.97; IMI 366417	Greece, Helianthus annuus	KP123876
CBS 102595; E.G.S. 45.100 (A. limoniasperaeT)	USA, Citrus jambhiri	AY563306
CBS 102596; E.G.S. 45.090 (A. citrimacularisT)	USA, Citrus jambhiri	KP123877
CBS 102598; E.G.S. 46.141 (A. citriarbustiT)	USA, Minneola tangelo	KP123878
CBS 102599; E.G.S. 44.166 (A. turkisafriaT)	Turkey, Minneola tangelo	KP123879
CBS 102600; E.G.S. 39.181; ATCC 38963 (A. toxicogenicaT)	USA, Citrus reticulata	KP123880
CBS 102602; E.G.S. 44.160 (A. perangustaT)	Turkey, Minneola tangelo	KP123881

CBS 102603; E.G.S. 45.011 (A. interruptaT)	Israel, Minneola tangelo	KP123882
CBS 102604; E.G.S. 45.007 (A. dumosaT)	Israel, Minneola tangelo	AY563305
CBS 109455	Canada, human arm tissue	KP123883
CBS 109803	Germany, human skin	KP123884
CBS 110027	Germany, human eye	KP123885
CBS 110977; E.G.S. 34.016; CBS 916.96; CBS 115616T	India, Arachis hypogaea	AY563301
CBS 112251 (A. arborescens)	Unknown, unknown	KP123887
CBS 112252 (A. tenuissima)	Unknown, unknown	KP123888
CBS 113013; CPC 4268 (A. tenuissima)	South Africa, Malus domestica	KP123889
CBS 113014; CPC 4260 (A. tenuissima)	South Africa, Malus domestica	KP123890
CBS 113015; CPC 4266 (A. tenuissima)	South Africa, Malus domestica	KP123891
CBS 113024; CPC 4334	South Africa, Minneola tangelo	KP123892
CBS 113025; CPC 4342	South Africa, Citrus clementina	KP123893
CBS 113054; CPC 4263 (A. tenuissima)	South Africa, Malus domestica	KP123894
CBS 115069; CPC 4254 (A. tenuissima)	South Africa, Malus domestica	KP123895
CBS 115152; HKUCC 9099	China, Psychotria serpens	KP123896
CBS 115188; CPC 4348	South Africa, Citrus clementina	KP123897
CBS 115190; CPC 4340	South Africa, Citrus sinensis	KP123898
CBS 115199; CPC 4327	South Africa, Minneola tangelo	KP123899
CBS 115200; CPC 4325	South Africa, Minneola tangelo	KP123900
CBS 115616; EGS 34.016; CBS 916.96; CBS 110977T	India, Arachis hypogaea	AY563301
CBS 116749	Netherlands, unknown	KP123901
CBS 117130	Italy, Arbutus unedo	KP123902
CBS 117143	Italy, Capsicum annuum	KP123903
CBS 118811; E.G.S. 35.158 (A. brassicinaeT)	USA, Brassica oleracea	KP123904
CBS 118812; E.G.S. 37.050 (A. daucifoliiT)	USA, Daucus carota	KP123905
CBS 118814; E.G.S. 44.048 (A. tomaticolaT)	USA, Solanum lycopersicum	KP123906
CBS 118815; E.G.S. 51.132 (A. tomaticolaR)	USA, Solanum lycopersicum	KP123907
CBS 118818; E.G.S. 31.032 (A. vacciniiT)	USA, Vaccinium sp.	KP123908
CBS 119115	Greece, Prunus sp.	KP123909
CBS 119399; E.G.S. 39.189 (A. postmessiaT)	USA, Minneola tangelo	KP123910
CBS 119408; E.G.S. 40.140 (A. herbiphorbicolaT)	USA, Euphorbia esula	JQ646410
CBS 119543; E.G.S. 12.160 (A. citricancriT)	USA, Citrus paradisi	KP123911
CBS 120829	Greece, Punica granatum	KP123912
CBS 121336; E.G.S. 37.005; ATCC 11680 (A. palanduiT)	USA, Allium sp.	KJ862259
CBS 121344; E.G.S. 45.003 (A. turkisafriaR)	Israel, Minneola tangelo	KP123913
CBS 121346; E.G.S. 45.056 (A. turkisafriaR)	South Africa, Minneola tangelo	KP123914
CBS 121348; E.G.S. 50.070 (A. platycodonisT)	China, Platycodon grandiflorus	KP123915
CBS 121454; E.G.S. 46.069 (A. destruensT)	USA, Cuscuta gronovii	JQ646402
CBS 121455; E.G.S. 50.078 (A. broussonetiaeT)	China, Broussonetia papyrifera	KP123916
CBS 121456; E.G.S. 50.080; HSAUP 9600197 (A. sanguisorbaeT)	China, Sanguisorba officinalis	KP123917
CBS 121492; HSAUP0207 (Ulocladium cucumisis)	China, Cucumis melo	KP123918
CBS 121544; E.G.S. 38.022 (A. caudataR)	USA, Cucumis sativus	KP123919
CBS 121547; E.G.S. 50.048 (A. yali-inficiensT)	China, Pyrus bretschneideri	KP123920
CBS 124277 (A. tenuissima)	Denmark, Prunus sp.	KP123921
CBS 124278 (A. tenuissima)	Denmark, Prunus sp.	KP123922
CBS 126071 (A. tenuissima)	Namibia, soil	KP123924
CBS 126072 (A. tenuissima)	Namibia, soil	KP123925
CBS 126908	USA, soil	KP123926

CBS 126910 (A. tenuis)	USA, soil	KP123927
CBS 127334	USA, soil	KP123928
CBS 127671; E.G.S. 52.121 (A. seleniiphilaT)	USA, Stanleya pinnata	KP123929
CBS 127672; E.G.S. 52.122 (A. astragaliT)	USA, Astragalus bisulcatus	KP123930
CBS 130254	India, human sputum	KP123931
CBS 130255	India, human sputum	KP123932
CBS 130258	India, human sputum	KP123933
CBS 130259	India, human sputum	KP123934
CBS 130260	India, human sputum	KP123935
CBS 130261	India, human sputum	KP123936
CBS 130262	India, human sputum	KP123937
CBS 130263	India, human sputum	KP123938
CBS 130265	India, human sputum	KP123939
Alternaria arborescens SC		
CBS 101.13; E.G.S. 07.022; QM1765 (A. geophilaT)	Switzerland, peat soil	KP123940
CBS 105.24; IHEM 3123 (A. alternata)	Unknown, Solanum tuberosum	KP123941
CBS 108.41; E.G.S. 44.087; ATCC 11892 (A. alternata)	Unknown, wood	KP123942
CBS 113.41; IHEM 3318 (A. alternata)	Unknown, Schizanthus sp.	KP123943
CBS 105.49 (A. alternata)	Italy, contaminant blood culture	KP123944
CBS 126.60; IMI 081622 (A. maritima)	UK, wood	JQ646390
CBS 750.68; LCP 68.1989 (A. tenuissima)	France, Phaseolus vulgaris	KP123945
CBS 102605; E.G.S. 39.128 (A. arborescensT)	USA, Solanum lycopersicum	AY563303
CBS 109730 (A. arborescens)	USA, Solanum lycopersicum	KP123946
CBS 112633; CPC 4244 (A. arborescens)	South Africa, Malus domestica	KP123947
CBS 115189; CPC 4345 (A. arborescens)	South Africa, Citrus clementina	KP123949
CBS 115516; CPC 4247 (A. arborescens)	South Africa, Malus domestica	KP123950
CBS 115517; CPC 4246 (A. arborescens)	South Africa, Malus domestica	KP123951
CBS 119544; E.G.S. 43.072 (A. cerealisT)	New Zealand, Avena sativa	KP123955
CBS 119545; E.G.S. 48.130 (A. senecionicolaT)	New Zealand, Senecio skirrhodon	KP123956
CBS 124281 (A. arborescens)	Denmark, Triticum sp.	KP123961
CBS 124282 (A. arborescens)	Denmark, Hordeum vulgare	KP123962
CBS 124283 (A. tenuissima)	Russia, Oryza sp.	KP123963
CPC 25266	Austria, Pyrus sp.	KP123965
Alternaria burnsii		
CBS 107.38; E.G.S. 06.185T	India, Cuminum cyminum	KP123967
CBS 110.50; MUCL 10012 (A. gossypina)	Mozambique, Gossypium sp.	KP123968
Alternaria gossypina		
CBS 102597; E.G.S. 45.114 (A. tangelonisT)	USA, Minneola tangelo	KP123978
CBS 102601; E.G.S. 45.017 (A. colombianaT)	Colombia, Minneola tangelo	KP123979
Alternaria jacinthicola		
CBS 133751; MUCL 53159T	Mali, Eichhornia crassipes	KP123984
Alternaria longipes		
CBS 113.35	Unknown, Nicotiana tabacum	KP123986
CBS 121332; E.G.S. 30.048R	USA, Nicotiana tabacum	KP123989

Abbreviation: (T): ex-type isolate; (R): representative isolate; Species names between parentheses refer to the former species name, Bold accession numbers are generated in other studies; np: no product.

Supplementary Table S3. PCR - RFLP profile of *Alt a1* gene region of *Alternaria alternata* isolates after digestion with two different restriction enzymes

Number of *Alternaria alternata* Isolate	*Alternaria alternata* Isolates	PCR Amplicon (bp)	Restriction Fragments (bp)		Restriction Pattern (A - D)
			*Hae*III (pb)	*Hinf*I (pb)	
31	CGJM 3112, CGJM 3028, CGJM 3125, CGJM 3105, CGJM 3064, CGJM 3070, CGJM 3017, CGJM 2965, CGJM 3114, CGJM 3101, CGJM 3069, CGJM 2984, CGJM 3062, CGJM 3063, CGJM 3127, CGJM 2979, CGJM 3161, CGJM 3116, CGJM 3171, CGJM 3150, CGJM 3149, CGJM 3173, CGJM 3546, CGJM 3547, CGJM 3551, CGJM 3115, CGJM 2973, CGJM 2974, CGJM 3124, CGJM 2954 , CGJM 3138	980	600	-	**A**
62	GJM 3027, CGJM 3108, CGJM 3079, CGJM 3030, CGJM 3142, CGJM 3054, CGJM 3055, CGJM 2956, CGJM 3139, CGJM 3041, CGJM 3029, CGJM 3130, CGJM 3071, CGJM 3072, CGJM 3168, CGJM 3104, CGJM 3002, CGJM 3003, CGJM 3145, CGJM 2975, CGJM 3153, CGJM 3007, CGJM 3169, CGJM 3008, CGJM 3009, CGJM 2952, CGJM 2962, CGJM 2963, CGJM 2970, CGJM 3129, CGJM 3001, CGJM 2995, CGJM 2992, CGJM 2968, CGJM 2971, CGJM 2994, CGJM 3011, CGJM 3018, CGJM 3014, CGJM 3015, CGJM 3013, CGJM 3019, CGJM 3035, CGJM 3020, CGJM 3077, CGJM 3000, CGJM 3004, CGJM 3073, CGJM 2983, CGJM 3046, CGJM 3177, CGJM 3010, CGJM 3118, CGJM 2998, CGJM 3120, CGJM 3128, CGJM 2978, CGJM 3100, CGJM 3099, CGJM 3083, CGJM 3165, CGJM 3162	980	310 + 200	-	**B**
122	CGJM 3107, CGJM 3053, CGJM 3119, CGJM 3057, CGJM 2955, CGJM 3109, CGJM 3126, CGJM 3120, CGJM 3154, CGJM 3067, CGJM 3091, CGJM 3084, CGJM 3160, CGJM 3123, CGJM 3061, CGJM 3005, CGJM 3047, CGJM 3111, CGJM 3051, CGJM 3024, CGJM 3050, CGJM 2964, CGJM 3033, CGJM 3093, CGJM 3049, CGJM 3037, CGJM 3052, CGJM 3048, CGJM 3131, CGJM 3164, CGJM 3045, CGJM 3065, CGJM 3039, CGJM 3166, CGJM 3141, CGJM 3034, CGJM 2977, CGJM 3096, CGJM 3068, CGJM 2993, CGJM 2999, CGJM 2982, CGJM 3023, CGJM 3022, CGJM 3106, CGJM 3012, CGJM 3075, CGJM 3038, CGJM 2980, CGJM 3086, CGJM 2976, CGJM 3016, CGJM 3170, CGJM 3117, CGJM 3156, CGJM 3152, CGJM 3157, CGJM 3146, CGJM 3133, CGJM 3151, CGJM 3172, CGJM 3136, CGJM 3135, CGJM 3134, CGJM 3148, CGJM 3147, CGJM 3174, CGJM 3158, CGJM 3159, CGJM 3175, CGJM 3082, CGJM 3088, CGJM 3094, CGJM 3092, CGJM 3176, CGJM 1A, CGJM 3081, CGJM 1B, CGJM 3167, CGJM 1C, CGJM1D, CGJM 1E, CGJM 1F, CGJM 3155, CGJM 1G, CGJM 3143, CGJM 1H, CGJM 1I, CGJM 3535, CGJM 3536, CGJM 3537, CGJM 3538, CGJM 3539, CGJM 3540, CGJM 3541, CGJM 3542, CGJM 3543, CGJM 3544, CGJM 3545, CGJM 3548, CGJM 3549, CGJM 3550, CGJM 3552, CGJM 3553, CGJM 3554, CGJM 3555, CGJM 3556, CGJM 3557, CGJM 3558, CGJM 3559, CGJM 3560, CGJM 3561, CGJM 3562, CGJM 2986, CGJM 3032, CGJM 3044, CGJM 3085, CGJM 3026, CGJM 3128, CGJM 2978, CGJM 3100, CGJM 3099	980	410	-	**C**
5	CGJM 3163, CGJM 2960, CGJM 2961, CGJM 2953, CGJM 2990	980	600+310+ 200	-	**D**

Table S3.
(*continued*)

Number of *Alternaria alternata* Isolate	*Alternaria alternata* Isolates	PCR Amplicon (bp)	Restriction Fragments (bp)		Restriction Pattern (E - F)
			*Hae*III (pb)	*Hinf*I (pb)	
2	CGJM 2985, CGJM 3040	980	410+180	-	
64	CGJM 2973, CGJM 2974, CGJM 3124, CGJM 2954,CGJM 2985, CGJM 3040, CGJM 3027, CGJM 3108, CGJM 3079, CGJM 3030, CGJM 3142, CGJM 3054, CGJM 3055, CGJM 2956, CGJM 3139, CGJM 3041, CGJM 3029, CGJM 3130, CGJM 3071, CGJM 3072, CGJM 3168, CGJM 3104, CGJM 3002, CGJM 3003, CGJM 3145, CGJM 2975, CGJM 3153, CGJM 3007, CGJM 3169, CGJM 3008, CGJM 3009, CGJM 2952, CGJM 2962, CGJM 2963, CGJM 2970, CGJM 3129, CGJM 3001, CGJM 2995, CGJM 2992, CGJM 2968, CGJM 2971, CGJM 2994, CGJM 3011, CGJM 3018, CGJM 3014, CGJM 3015, CGJM 3013, CGJM 3019, CGJM 3035, CGJM 3020, CGJM 3077, CGJM 3000, CGJM 3004, CGJM 3073, CGJM 2983, CGJM 3046, CGJM 3177, CGJM 3010, CGJM 3118, CGJM 2998, CGJM 3120, CGJM 2986, CGJM 3032, CGJM 3044	980	-	410+180	**E**
158	CGJM 3112, CGJM 3028, CGJM 3125, CGJM 3105, CGJM 3064, CGJM 3070, CGJM 3017, CGJM 2965, CGJM 3114, CGJM 3101, CGJM 3069, CGJM 2984, CGJM 3062, CGJM 3063, CGJM 3127, CGJM 2979, CGJM 3161, CGJM 3116, CGJM 3171, CGJM 3150, CGJM 3149, CGJM 3173, CGJM 3546, CGJM 3547, CGJM 3551, CGJM 3115, CGJM 2973, CGJM 2974, CGJM 3124, CGJM 2954 , CGJM 3138, CGJM 3163, CGJM 2960, CGJM 2961, CGJM 2953, CGJM 2990, CGJM 3107, CGJM 3053, CGJM 3119, CGJM 3057, CGJM 2955, CGJM 3109, CGJM 3126, CGJM 3120, CGJM 3154, CGJM 3067, CGJM 3091, CGJM 3084, CGJM 3160, CGJM 3123, CGJM 3061, CGJM 3005, CGJM 3047, CGJM 3111, CGJM 3051, CGJM 3024, CGJM 3050, CGJM 2964, CGJM 3033, CGJM 3093, CGJM 3049, CGJM 3037, CGJM 3052, CGJM 3048, CGJM 3131, CGJM 3164, CGJM 3045, CGJM 3065, CGJM 3039, CGJM 3166, CGJM 3141, CGJM 3034, CGJM 2977, CGJM 3096, CGJM 3068, CGJM 2993, CGJM 2999, CGJM 2982, CGJM 3023, CGJM 3022, CGJM 3106, CGJM 3012, CGJM 3075, CGJM 3038, CGJM 2980, CGJM 3086, CGJM 2976, CGJM 3016, CGJM 3170, CGJM 3117, CGJM 3156, CGJM 3152, CGJM 3157, CGJM 3146, CGJM 3133, CGJM 3151, CGJM 3172, CGJM 3136, CGJM 3135, CGJM 3134, CGJM 3148, CGJM 3147, CGJM 3174, CGJM 3158, CGJM 3159, CGJM 3175, CGJM 3082, CGJM 3088, CGJM 3094, CGJM 3092, CGJM 3176, CGJM 1A, CGJM 3081, CGJM 1B, CGJM 3167, CGJM 1C, CGJM 1D, CGJM 1E, CGJM 1F, CGJM 3155, CGJM 1G, CGJM 3143, CGJM 1H, CGJM 1I, CGJM 3535, CGJM 3536, CGJM 3537, CGJM 3538, CGJM 3539, CGJM 3540, CGJM 3541, CGJM 3542, CGJM 3543, CGJM 3544, CGJM 3545, CGJM 3548, CGJM 3549, CGJM 3550, CGJM 3552, CGJM 3553, CGJM 3554, CGJM 3555, CGJM 3556, CGJM 3557, CGJM 3558, CGJM 3559, CGJM 3560, CGJM 3561, CGJM 3562, CGJM 3085, CGJM 3026, CGJM 3128, CGJM 2978, CGJM 3100, CGJM 3083, CGJM 3165, CGJM 3162	980	-	400+200+110	**F**
